# Meaningful Learning Experiences in Everyday Life During Pandemics. A Qualitative Study

**DOI:** 10.3389/fpsyg.2021.670886

**Published:** 2021-05-07

**Authors:** Irene González-Ceballos, Montserrat Palma, Josep Maria Serra, Moisès Esteban-Guitart

**Affiliations:** Department of Psychology, Institute of Educational Research, University of Girona, Girona, Spain

**Keywords:** learning, education, digital life, COVID-19 pandemic, qualitative research

## Abstract

The COVID-19 pandemic has drastically changed the lives of people all over the world. In particular, an unprecedented educational crisis has occurred due to the circumstances of physical distancing and remote learning. This article focuses specifically on the meaningful learning experiences in the everyday lives of adolescents during the pandemic. 72 meaningful learning experiences were identified from 11 participants who recorded their specific learning experiences for a week by a means of a journal recorded by themselves. A content analysis was undertaken in order to identify the ecology (what, how, where, and who with) of the different learning experiences. The results show a prevalence of personal and conceptual learning, a presence of both formal and specifically informal, everyday activities among the meaningful learning experiences detected, the importance of peers, teacher and “learning experiences while alone,” and the use of digital technologies as learning resources; they also reveal the assistance of others in the learning process. The main contribution of this study illustrates how students in everyday life during pandemics are involved in a whole range of different activities both at school and at home.

## Introduction

In recent decades, the impact information and communication technologies have on the transformation of both learning processes and educational practices has been documented (Jenkins, 2009; Coll, 2013; González-Patiño and Esteban-Guitart, 2014; Bender and Peppler, 2019; Gee and Esteban-Guitart, 2019). In particular, recently, different studies have documented the impact of remote education, as well as the emergence of hybrid models (online-offline), on educational inequalities, as well as teaching and learning processes (Arora and Srinivasan, 2020; Iglesias et al., 2020; Jena, 2020; Paudel, 2021). However, this literature did not address the impact of pandemics on learning experiences of the young. This article aims to contribute to the existing literature by considering the impact that the COVID-19 pandemic has on the meaningful learning experiences of young people from different socioeconomic and sociocultural conditions.

We understand meaningful learning experiences as being those that, due to their cognitive-emotional impact, the learner identifies as being especially relevant. Additionally, the learner grants a particular meaning to the set of recognized learning experiences achieved throughout the day, beyond the bounds of context and place where the learning experiences occur (Esteban-Guitart, 2016; Esteban-Guitart et al., 2017). According to Esteban-Guitart (2016) meaningful learning experiences mean “those that the learner selects and chooses from his/her prior learning experiences, for their positive or negative impact. These experiences are the most relevant from the learner’s point of view, for whatever reason, and are connected to their needs or interests” (p. 52).

Previous research suggests that educational times and spaces have both been modified and that this is mainly due to the porosity of digital practices and cultures. In this sense, we speak not only of learning throughout life but also life-wide: the result of participation in different contexts, situations and daily educational practices, both social and in the community (Esteban-Guitart et al., 2018). Based on the Bronfenbrenner’s ecological systems theory (Bronfenbrenner, 1979), the notion of learning ecologies, in this sense, considers the set of physical and/or virtual activities, the help, collaboration and guidance of other people, as well as the different resources, inside and outside the school education context, as potential opportunities for learning available to a learner (Barron, 2004).

Taking the very notion of “learning ecologies” as a reference (Barron, 2004, 2006), Coll (2013) argues that we are facing a profound revision of the fundamental parameters that characterize educational practice (where, when, what, who with, why and how we learn). From a model focused on universal schooling, belonging to the twentieth century, we are now in a moment of transition toward distributed and interconnected emerging models. In this sense, we speak of “local learning ecosystems” (Hannon et al., 2019) to refer to a great multiplicity of interconnected educational scenarios and agents, linked to the development of basic competencies or skills for the 21st century, through participation in affinity groups or communities of practice, in different physical and digital mediums, as well as in distinct narrative formats (DiGiacomo D. et al., 2018; Lacasa, 2018).

In a previous study, the importance of informal situations and practices was identified, as generators of even school-type learning (aspects related to the science or history curriculum, for example); the importance of the peer group, and the “self-taught” situations—learning that one claims to have undertaken alone—, as well as in digital format (for example, YouTube, social networks, Internet content search) and from the participation in communities of affinity or interest, such as a Facebook group, or online gamers, as geography/format of a large part of the meaningful learning experiences identified in adolescents aged 15 and 16 (Esteban-Guitart et al., 2017).

These results are in tune with the literature linked to “connected learning” according to which, a large part of learning is currently generated from the link or connection between a certain interest and curricular, professional or civic opportunities, through collaboration and support from others, forexample through social networks (DiGiacomo D. K. et al., 2018; González-Patiño and Esteban-Guitart, 2019; Esteban-Guitart et al., 2020a).

However, often these learning experiences that take place in non-formal or informal spaces of activity, are neither taken advantage of, nor linked to, the curricular type learning that takes place in school. “The majority of young people do not find ways to connect learning in their online affinity networks with in-school, civic, or career-relevant opportunities” (Ito et al., 2019, p. 2).

In any case, it seems clear that the opportunities and sources of learning today transcend the walls and borders of the school educational context and practice, as digital mobile devices allow access, construction and exchange of knowledge, skills, and competences. What Jenkins (2009) refers to as the concept of “participatory cultures” characterized by the ability to produce and exchange content and experiences through different media such as amateur videogame design, films or songs shared through YouTube, blogs, Facebook, Instagram, or other social and digital media.

The aim of the study presented here is to identify, and analyze, meaningful learning experiences experienced over the course of a week by 16 and 17-year-old adolescents during the COVID-19 pandemic situation in order to illustrate the potential impact of the pandemic situation on learning processes and ecologies.

## Materials and Methods

With the aim of achieving the aims of the research, and in accordance with the unit of analysis described in the introduction, meaningful learning experiences, a qualitative approach was used in the consideration of the identification and analysis of the subjectivity as a proposal for the generation of knowledge (Mruck and Breuer, 2003). In particular, and in the same line as previous research (Esteban-Guitart et al., 2017), a content analysis, described below, was carried out.

### Participants

An intentional sample, deliberately chosen, of 11 participants was selected from a first-year high school class of 28 students from a state school in a neighborhood characterized by its high sociocultural diversity in Girona, Catalunya, Spain. The sample was composed of five boys and six girls between 16 and 17 years of age, balancing gender distribution. Of the total, six are of local origin (Catalan, Spanish), while five students come from abroad (two from Honduras, one from Colombia, one from Bolivia, and one from Morocco). The purpose was to reflect the diversity of both the school and context of the region. Table 1 describes the sociodemographic characteristics of the participants. For reasons of confidentiality, a code was assigned to the different participants.

**TABLE 1:** Sociodemographic characteristics of the sample.

**Code**	**Age**	**Sex**	**Mother tongue**	**Origin**	**Parents’ work**
BCT001	16	Female	Castilian	Spain	Delivery man/Child educator
BCT002	17	Female	Catalan	Spain	Engineer/Sales representative
BCT003	17	Male	Catalan	Spain	Marketing Coordinator/R&D Biotech
BCT004	17	Male	Castilian	Colombia	Slaughterhouse operative/Cook
BCT005	17	Male	Castilian	Bolivia	Builder/Caregiver
BCT006	16	Female	Castilian	Spain	Cleaner/Rebuilding company
BCT007	16	Male	Castilian	Honduras	Carpenter/Baker
BCT008	16	Male	Catalan	Spain	Security guard/Careers advisor
BCT009	16	Female	Arabic	Morocco	Unemployed
BCT010	16	Female	Catalan and Castilian	Spain	Government employees
BCT011	16	Male	Castilian	Honduras	Truck driver/Cleaner

Table 2 additional data regarding the participants in relation to their learning ecologies (Barron, 2004), specifically the availability, or otherwise, of an Internet connection, together with available devices and usual practices carried out during the week as well as going to high school.

**TABLE 2:** Some characteristics of the participants’ learning ecologies.

**Code**	**Age**	**Internet connection (devices available)**	**After-school activities**
BCT001	16	Yes (mobile phone, laptop, and game console)	Physical exercise at home.
BCT002	17	Yes (mobile phone, shared computer, shared laptop, and game console)	Modern dance instructor in after-school activities.
BCT003	17	Yes (mobile phone, laptop, and game console)	Sport: rugby
BCT004	17	Yes (mobile phone and laptop)	Sport: soccer
BCT005	17	Yes (mobile phone, laptop, game console, and television)	Nothing reported
BCT006	16	Yes (mobile phone and shared laptop)	Mathematics class (school tutoring)
BCT007	16	Yes (mobile phone, shared laptop, game console, and tablet)	Sport: soccer
BCT008	16	Yes (mobile phone and laptop)	Cycling and physical exercise at home
BCT009	16	Yes (mobile phone, shared laptop, television, and game console)	Cycling
BCT010	16	Yes (mobile phone, laptop, and television)	Attending dance classes
BCT011	16	Yes (mobile phone, laptop, and game console)	Nothing reported

All participants have an Internet connection, as well as personal mobile phones and seven personally owned laptop computers, in four cases shared with either siblings or parents. With regard to after-school activities, what stands out are sports and physical activities. Two participants did not report doing any after-school activity during the week (see Table 2).

### Instrument

**FIGURE 1:**
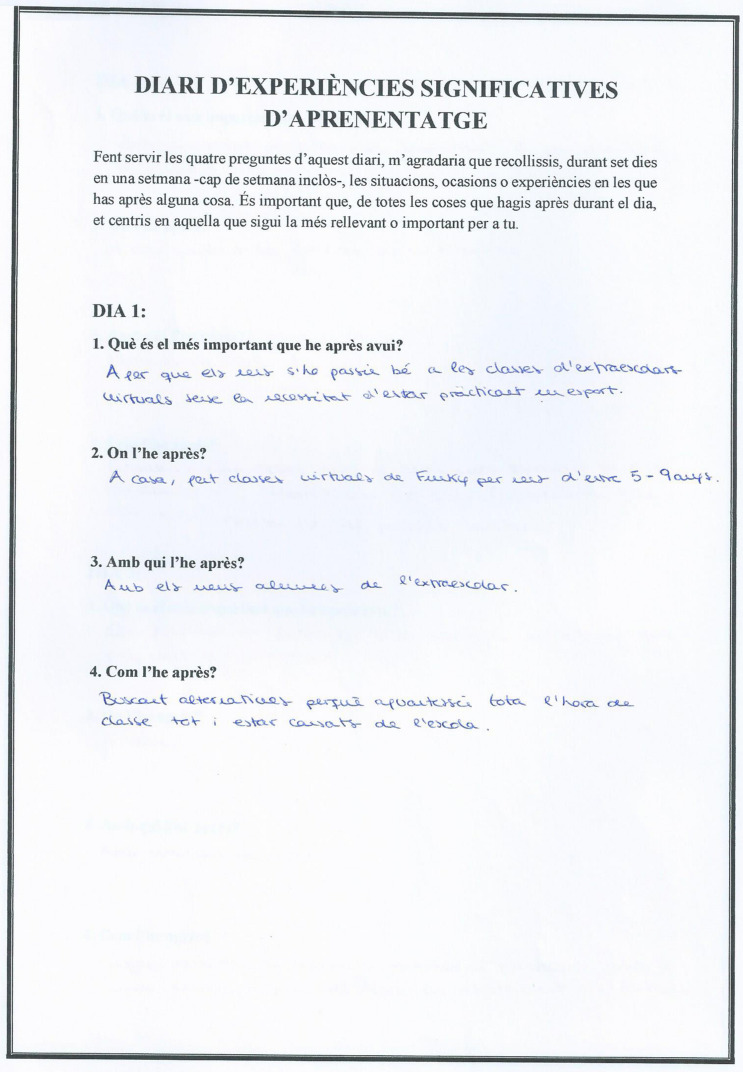
Example of a personal diary of meaningful learning experiences.

With the aim of identifying meaningful learning experiences, an adaptation of the personal journal of meaningful learning experiences proposed by Esteban-Guitart et al. (2017) was used. The original version consisted of five questions, in our version we used four questions that the participants had to answer at the end of the day for a week (see Figure 1). Specifically, the data was collected between Monday, 25th January and Sunday, 31st January, 2021. The questions were: (a) What is the most important thing you learned today? (b) Where did you learn it? (c) Who with? (d) How did you learn it? The instructions were: “Using the four questions in this diary, I would like you to collect, over 7 days of a week—weekend included-, the situations, occasions or experiences where you learned something. It is important, that of all the things learned throughout the day, you focus on the one that is most relevant or important to you.”

With the aim of identifying some of the characteristics of the participants’ learning ecologies, information was collected *via* an on-line questionnaire. This information consisted of: the availability or otherwise of an internet connection, the digital devices available; as well as the after-school activities carried out during the week (see Table 2).

### Procedure

Firstly, the study was approved by the research ethics and biosafety committee (CEBRUdG) of the University of Girona. Next, the research proposal was presented to the director of the school, and to the classroom teacher of the participants. After its approval, a sample of 11 participants was taken from the class group. They were contacted and informed of the purpose of the research, and authorization was sought to participate in the study based on informed consent. Once the instrument, a personal diary of meaningful learning experiences, was provided by the research team, the participants filled it out during the week of 25th–31st January, 2021. Finally, an on-line questionnaire was administered to each participant to identify the availability or otherwise of digital devices, the availability of an Internet connection, as well as the activities carried out during the week. During this period, classes were physically attended in the formal educational context with mask and hygiene measures, although 1 day a week, on Wednesday, classes were not attended in person and were held on-line. On the other hand, there was a situation of semi-confinement, since at that time there were measures affecting bars, cultural facilities and shops in the region of Catalonia. Specifically, non-essential shops were ordered to close at weekends, as well as shopping centers of more than 400 m^2^. On the other hand, restaurants and cafes were allowed to open but only between the times of 7:30 a.m. to 9:30 a.m. and 1 p.m. to 3:30 p.m. There was a limitation to interior capacity of 30%. There was also confinement on a municipal level in that entering and leaving Girona was restricted except for a justified reason. This measure affected the mobility of the population. One of the exceptions was that of attending school. However, all university lectures were online and face-to-face classes at the university were suspended.

### Data Analysis

In order to analyze the empirical data obtained, thematic content analysis procedure was used with a deductive-inductive category system procedure in a round-trip iterative process between the data and the initial categories that were enriched and modified from the analysis carried out (Vaismoradi and Snelgrove, 2019). In particular, an answer was found to the research question related to the characterization of the ecology (what, where, how, who with) of meaningful learning experiences. To do this, we initially based our research on the *a priori* categories developed by Esteban-Guitart et al. (2017) from the parameters of the new learning ecology described by Coll (2013). However, the category “what” was added, which was not analyzed in the study by Esteban-Guitart et al. (2017). It was decided to include this category because although it is contemplated in the parameters of the new learning ecology (Coll, 2013), it was not included in the research undertaken by Esteban-Guitart et al. (2017). This was considered to be a limitation in itself as it was not possible to obtain any analysis about the content of the meaningful learning experiences identified. In order to operationalize this category, the conceptual, procedural, and/or personal-identity learning contents were used. To this end, the learning types described by García-Romero et al. (2018), Lalueza and Macías-Gómez-Estern (2020), and Macías-Gómez-Estern et al. (2019) in research on the evaluation of the learning service by university students is used. In order to readjust the previous categories inductively based on the data obtained, inclusion criteria were introduced. Table 3 shows these categories, codes and inclusion criteria finally used in the study.

**TABLE 3:** Categories, codes and inclusion criteria used.

**Categories**	**Analysis codes**		**Inclusion criteria**
	Conceptual		When the participant describes a theoretical type of learning, of facts and concepts. It includes the ability to identify, recognize, describe and compare objects, events or ideas.
What?	Procedural		When the participant describes learning based on actions and operations, either in practice or mentally. A set of ordered and completed actions, that is to say, aimed at the achievement of a goal. These include ability, technique, methods and strategies.
	Personal		When the participant describes learning related to values, attitudes or rules in relation to their own subjectivity. It includes beliefs, sentiments, preferences, actions and declarations of intentions.

		Educational institution	When the participant describes learning as a result of participation in a formal educational institution and is physically inside the institution.
	Formal	From home	When the participant describes learning as derived from participation in a formal educational institution, but physically he or she is at home. This includes online classes and other activities derived from academic work or study.
Where?	Not formal		When the participant describes learning as the fruit of an organized, planned educational activity undertaken outside the structure of the formal system. This includes activities that are not explicitly educational, but that contain components to support the learning process (training courses, free-time or sporting activities, extracurricular activities).
	Informal		When the participant describes spontaneous learning situations outside traditional educational institutions.

	Alone Teacher		When the participant reports learning while alone, without any other person present. This includes teachers as well as other professionals who are involved in formal teaching and learning activities, such as librarians.
Who with?	Peer group		Classmates, partner, and/or friends.
	Relatives		Legal guardians, fathers, mothers, brothers, sisters, uncles, cousins, grandparents.
	Other		Social or community agents.

	Cultural mediation		Traditional format: physical mediator such as a book, magazine, manual, etc.
			Digital format: physical mediator a digital resource or medium such as the Internet through a mobile phone, computer.
How?	Without cultural mediation		When the participant describes learning as a consequence of a reflective process without interaction with or use of any cultural artifact.
	Social mediation		Learning is described as a result of social interaction.
	Without social mediation		Learning is described as a result of personal work-reflection, without the explicit involvement of other people.

In relation to the coding process, the codes that appear in Table 3 have been assigned to the text segments of the diaries written by the participants about their meaningful learning experiences. This analysis allows collection of the frequencies of citations associated with the different categories and analysis codes.

“High intercoder reliability” (ICR) (Burla et al., 2008) is used in qualitative content analysis carried out for ensuring concordance in data analysis. In particular, transcripts were coded independently by two researchers from the categories, codes and inclusion criteria used (see Table 3) by two researchers, and ICR was calculated. A resulting kappa value of 0.91 can be regarded as solid.

## Results

A total of 72 meaningful learning experiences were identified as a result of the seven experiences that each participant has selected on each of the 7 days of the week; With the exception of BCT007 and BCT009, who stated, on 3 days in the first case and on 2 days in the second case, that they had not learned anything relevant throughout the day.

Regarding the content category of the meaningful learning experiences (the “What?”), the identity-personal subcategory stands out (with 32 citations), followed by conceptual (with 30) and procedural (with 10). Regarding the “Where?”, the formal educational context stands out, either at the educational institution (24 associated citations) or at home (with six citations) but in activities, such as homework, extension of time, and homework. However, most meaningful learning experiences originated in informal contexts or situations. In relation to the “Who with?” The code that obtains a greater association of citations is “Alone,” followed by the peer group and the teacher. To a lesser extent with the family. Finally, at the level of “How?” what stands out is the consideration of learning without any type of cultural mediation, without the use of artifacts; however, in the case of the use of artifacts, the digital format stands out. Finally, it is worth highlighting social interaction as a generator of a large part of the meaningful learning experiences, compared to self-learning (see Table 4).

**TABLE 4:** Citations associated with the different categories and analysis codes.

**Categories**	**Analysis codes**		**Number of citations**	**Example**
What?	Conceptual		30	“How (radio/TV/mobile phone) waves work” (BCT003, day 2)
	Procedural		10	“Make wireless earphones work” (BCT002, day 7)
	Identity-Personal		32	“That the person who loves you most is not the person who most tells you so” (BCT001, day 6)

Where?	Formal	Educational institution	24	“I learned what logic is and how to identify formal fallacies in philosophy class, at high school” (BCT010, day 4)
		At home	6	“I learned a lot about plastic elastomers for a class project at home” (BCT010, day 6)
	Not formal		3	“At home, doing virtual Funky dance classes for 5–9-year-old children” (BCT002, day 1)
	Informal		39	“I learned at home that there are non-parliamentary political parties” (BCT008, day 5)

Who with?	Alone		25	“Titanium is a very practical, useful material but the way to get it is very expensive, I learned this at home, alone, looking for information” (BCT005, day 3)
	Teacher		18	“The teacher taught me new calculator functions in class” (BCT009, day 5)
	Peer group		19	“I learned with my friends how to improve my playing skills in a competitive game” (BCT003, day 4)
	Relatives		7	“With my family, I learned how my parents drive” (BCT003, day 7)
	Other		3	“At the doctor’s” (BCT011, day 1)

How?	Cultural mediation		3 (traditional format)	“In class I learned how to do trigonometric equations with the maths book” (BCT008, day 4)
			18 (digital format)	“Looking on the Internet how to install an application on the computer without having to pay and with no kind of virus” (BCT003, day 3)
	Without cultural mediation		51	“By listening in class, I learned that we have a set of chromosomes, in total 46” (BCT005, day 4)
	Social mediation		47	“In the street with friends, talking and thinking together, I learned the value of telling the truth and keeping promises” (BCT004, day 1)
	Without social mediation		25	“I learned that physical education is really necessary, after so much time without activity I thought about that” (BCT009, day 4)

For the purposes and context of this research, and of this monographic issue, the presence of COVID is highlighted in four of the meaningful learning experiences reported. As can be seen in Table 5, these are current issues in the pandemic period in which the study was carried out, for example, vaccines.

**TABLE 5:** Meaningful learning experiences associated with COVID-19.

BCT006 (day 3) WHAT: “COVID vaccines carry mRNA of itself” WHERE: “In online class” WHO WITH: “With a video of a medical scientist” HOW: “Looking at the video that they showed us in class”

BCT002 day 2 WHAT? “That in this country politics is more important than health” WHERE? “At home, looking at the television news” WHO WITH? “TV3 news” HOW? “Listening to how they said they were going to have an election in the middle of a pandemic and seeing how they apply the measures that they want without listening to health workers”

BCT006 day 2 WHAT? “Mental health depends on knowing how to manage the seven emotions that we may have during the day” WHERE? “At school” WHO WITH? “With the chemistry teacher” HOW? “We were in class talking about coronavirus and the different ways how it affects society”

BCT010 day 7 WHAT? “That the confinement and the different restrictions have affected each person in different ways and that means our priorities change” WHERE? “At home” WHO WITH? “With my sister” HOW? “Because she explained it to me”

Taken as a whole, meaningful learning experience linked to identity-personal aspects (for example, linked to the organization of tasks, values or aspects linked to knowledge about oneself) have been derived from informal situations and contexts of life and activity. For example, BCT002 (day 5) claims to have learned that doing things in advance (for example homework) frees up time for leisure. The participant says she learned it alone, at home, specifically “doing all the homework she had for the following week in order to make the most of the weekend, even though we can’t go out much.” Meanwhile the curricular-conceptual type learning takes place in the formal sphere. However, conceptual learning carried out at home is also highlighted, acting as a support and extension of school activities, as well as derived from informal situations in seven of the total experiences with formal content (30). For example, derived from a chat with friends, BCT010 claims, on the second day, to have learned the equation of the trajectory of movement. However, the majority of conceptual learning took place in the school and was basically facilitated by the teacher. While learning carried out in informal life situations or practices is associated with situations in which the learner claims to be alone, or with peers—basically connected through digital devices. In relation to this, the use of social networks such as Instagram, video games, or search engines such as Google stand out. For example, BCT008, on the seventh day, claims to have learned to play 1-min games of chess with the computer, through an online chess game; or BCT007 (day 2) claims to have learned with Instagram that the first love one must receive is one’s own love. In reality, a large part of the situations considered self-learning are characterized by the use of digital devices. For example, BCT003, on the third day, learned how to install an application on the computer without paying by searching for information on the Internet.

## Discussion

The exceptional situation, derived from the COVID-19 pandemic, has had an impact on different aspects of people’s daily lives. The aim of the study presented here was to identify different meaningful learning experiences in the pandemic situation. In a previous study, the importance of informal learning situations and contexts was identified, as well as the importance of social and digital media as spaces for interaction and learning (Esteban-Guitart et al., 2017). This study is in tune with the previous study, despite the fact that the formal educational context also appears as relevant, perhaps as it is the main activity of young people, as well as being at home, as they are in a moment of semi-confinement; with restrictions in shops, bars and cafes and mobility. In fact, the study illustrates how the learning processes took place either in high school or at home. Highlighting learning undertaken alone. A situation that can also be explained due to the social restrictions of personal contact and mobility. However, a large number of the experiences have been categorized as resulting from social mediation (47) as opposed to those without social mediation (25), since the peer group at school and through their contact with digital devices is observed as an element which is highlighted from many of the meaningful learning experiences identified. This is in agreement with the work of DiGiacomo D. K. et al. (2018) that shows the importance of social and material conditions in the development of interests and learning objectives in adolescents and young people.

Compared with the study by Esteban-Guitart et al. (2017), the aforementioned difference in terms of the presence of the formal environment in meaningful learning experiences (not found in that study, and considerably significant in this one), the distribution and importance of the peer group, and the situations of “Being alone” are found to have the same trend. Although in this study, these situations increase proportionally, perhaps due to the pandemic situation, as well as the presence and importance of the teacher in such learning; this aspect was not identified in the previous study. Finally, in the “how,” the presence of the digital format or tools and practices also stands out. It is important to highlight here that all the participants reported having an Internet connection as well as mobile phones and either shared, or unshared, laptop computers.

Regarding critical considerations of the study undertaken, it is necessary to consider that the qualitative nature of the study, involving 11 adolescents and young people, prevents us from reaching conclusions and generalizing the results to other contexts and situations. In addition, the categories of analysis should be reconsidered in future works, since due in large part to the porosity of digital media and devices, it is difficult to identify the border of the contexts. For example, BCT011, on the first day, claimed to have learned that they had Raynaud’s Syndrome. He learned it, in fact, in a multiplicity of situations and contexts, after, as he describes it, talking with his doctor, searching on the Internet, consulting with his family and friends, and also especially from the “My Health” digital application. In the same way, learning from the formal, school environment, although it may begin in the context of the high school, continues through the internet, with the peer group and especially in this study described here, at home.

This consideration leads us to problematize the notion of context itself, understood as a physical and/or virtual environment, more or less defined in time and space, in which the learner participates directly, adopts different roles, activities and interpersonal relationships (Bronfenbrenner, 1979), toward procedural considerations that take into account the hybrid and porous nature of learning situations that in fact question the traditional separation between the formal and informal sphere (Jornet and Erstad, 2018; Esteban-Guitart et al., 2020b; González-Patiño and Esteban-Guitart, 2021). In any case, future research should trace, through for example, life stories and case studies, the parameters considered here under the metaphor of the new learning ecology (Coll, 2013). The aforementioned research would allow us to go into greater depth regarding both the conditions and the characteristics of the learning experiences, as well as to overcome certain limitations of the categories used, for example in relation to the “how?” category. This is because by reducing this category to the mere presence or absence of cultural or social mediation does not allow for an in-depth documentation of the process of acquisition and development of the learning experience described.

## Conclusion

The aim of the research was to examine the meaningful learning experiences throughout the unusual situation experienced during the COVID-19 pandemic. A content analysis was undertaken, following the same line of previous research (Esteban-Guitart et al., 2017). This made it possible to document 72 meaningful learning experiences of 11 adolescents from 16 to 17 years old. The meaningful learning experiences were collected for a week by means of a personal diary. Using the “new learning ecology” (Coll, 2013): what, where, who with and how learning happens, the content analysis was carried out. Concerning “what,” the results show a prevalence of learning experiences related to subjectivity (values, attitudes, beliefs, sentiments, and preferences) and conceptual learning (learning of facts and concepts); concerning where, there is a presence of both formal and informal learning experiences; concerning “who with,” the most frequent learning experiences are with peers, with teachers and alone; and concerning “how,” the results show the relevance of digital technologies as learning resources. The main contribution of this research consists in the empirical documentation of the aforementioned parameters in the context of a pandemic. However, as this is a rather unrepresentative sample, in no way is it intended to make a generalization of the results found. On the other hand, a case study would allow for a greater depth of documentation of the learning experiences as well as their characteristics and conditions. Regarding practical applications, it should be noted that the learning experiences need pedagogical consideration, not just taking into account where they have originated, as this throws light on the situational and distributional character of the learning experiences. This aspect follows the line of previous research on the analysis of different situations in formal contexts, non-formal contexts and informal contexts as generators of meaningful learning experiences (Barron, 2004; Esteban-Guitart, 2016; Esteban-Guitart et al., 2017, 2018; DiGiacomo D. et al., 2018; Bender and Peppler, 2019).

## Data Availability Statement

The raw data supporting the conclusions of this article will be made available by the authors, without undue reservation.

## Ethics Statement

The studies involving human participants were reviewed and approved by the research ethics and biosafety committee (CEBRUdG) of the University of Girona. Written informed consent to participate in this study was provided by the participants’ legal guardian/next of kin.

## Author Contributions

ME-G conceptualized the research idea and planned the study. IG-C carried out the collecting data. MP and JS contributed to data analysis. ME-G, IG-C, MP, and JS contributed to the interpretation of the results. ME-G and IG-C wrote the manuscript. All authors provided critical feedback and helped shape the manuscript.

## Conflict of Interest

The authors declare that the research was conducted in the absence of any commercial or financial relationships that could be construed as a potential conflict of interest.
